# Potent Anticancer Effects of Bioactive Mushroom Extracts (*Phellinus linteus*) on a Variety of Human Cancer Cells

**DOI:** 10.14740/jocmr1996w

**Published:** 2014-11-19

**Authors:** Sensuke Konno, Kevin Chu, Nicholas Feuer, John Phillips, Muhammad Choudhury

**Affiliations:** a Department of Urology, New York Medical College, Valhalla, New York, USA

**Keywords:** Anticancer, Mushroom, PL-fractions, Apoptosis, Human cancer cells

## Abstract

**Background:**

Although several therapeutic options are currently available for patients with various cancers, the outcomes are often disappointing and a more effective modality needs to be promptly established. We have been exploring an alternative approach using natural agents and two bioactive mushroom extracts isolated from *Phellinus linteus* (PL), namely PL-ES and PL-I-ES, were of our interest. As anticancer effects of similar extracts have been reported in several cancers, we investigated whether PL-ES and PL-I-ES might have such anticancer activities on a variety of human cancer cells *in vitro*.

**Methods:**

Ten different types of human cancer cell lines, including three metastatic prostate, bladder, kidney, lung, breast, stomach, liver, and brain cancer cells, were employed and tested with PL-ES or PL-I-ES. Cell growth/viability, exertion of oxidative stress, and induction of apoptosis were assessed by MTT (3-[4,5-dimethylthiazol-2-yl]-2,5-diphenyl-tetrazolium bromide) assay, lipid peroxidation (LPO) assay, and specific enzymatic assay, respectively.

**Results:**

PL-ES (100 µg/mL) exhibited potent anticancer activity, resulting in a significant (40-80%) growth reduction in all 10 cancer cells at 72 hours. PL-I-ES (100 µg/mL) was effective on only four cancer cells but its higher concentration at 250 µg/mL led to a significant (25-90%) growth reduction in seven cancer cells. LPO assays indicated that such a significant growth reduction by PL-ES (100 µg/mL) or PL-I-ES (100 or 250 µg/mL) could result from cell death due to a cytotoxic effect of oxidative stress (through free radicals). Moreover, enzymatic assays for caspase-3 (Csp-3) and caspase-9 (Csp-9), the pro-apoptotic regulators, showed that both enzymes were significantly activated by PL-ES or PL-I-ES, indicating that cell death due to oxidative stress was more likely associated with apoptosis.

**Conclusions:**

The present study shows that both PL-ES and PL-I-ES indeed have anticancer effects on a variety of cancer cells, although PL-ES appears to be more potent than PL-I-ES. Such an anticancer effect is presumably attributed to oxidative stress, which will ultimately lead to apoptosis. Therefore, these two bioactive mushroom extracts may have clinical implications in a more effective therapeutic option for a variety of human malignancies.

## Introduction

The leading cancer types in the United States (in 2013) include prostate, breast, lung, colon, melanoma (skin), bladder, kidney and so forth [1]. However, the death rates (mortalities) from various cancers vary from cancer to cancer and some of them are rather deadly. For instance, a mortality of lung cancer is about 70% (160,000 deaths of 230,000 patients) and those of pancreatic and liver cancers are about 85% (39,000 of 45,000) and about 71% (22,000 of 31,000), respectively, although their incidences are not as high as lung cancer [1]. Moreover, a mortality of colon cancer is about 50% but as many as about 51,000 deaths, while about 40,000 and about 30,000 deaths (with about 17% and about 13% death rates, respectively) come from breast and prostate cancers, respectively [1]. Hence, pancreatic, liver, and lung cancers are the most deadly cancers (with the high mortalities) but the numbers of deaths from breast and prostate cancers (with the relatively low mortalities) are as high as or even higher than these cancers. It is not the “numbers” that matter but all cancers are equally dismal and deadly. It is thus true that cancer is indeed a leading cause of death in the US as well as worldwide, making it a global health problem [2].

Several conventional therapeutic options for cancers, such as surgery, internal/external radiotherapy, hormone therapy, chemotherapy, immunotherapy, etc., are currently available. Although they may have the initial good response rates, most deaths are attributed to a subsequent relapse or recurrence of the disease developed in large numbers of patients [3]. For instance, although endoscopic transurethral resection is often performed as a primary therapy to remove superficial bladder tumors, 60-70% of patients will recur and about 10% will progress to muscle invasive disease within 5 years [4], becoming untreatable and fatal. Similarly, after complete resection of kidney tumor (by nephrectomy), 20-30% of patients will progress to a metastatic disease with the 5-year survival rate of < 10% [5]. In addition, chemotherapy is yet currently one of the most common therapeutic options, although some outcomes are encouraging but most of them have been rather disappointing or unsatisfactory with severe side effects [6]. Other viable options are also implemented but again few of them are working. Thus, safer and more effective treatments for these cancers are urgently and sturdily demanded.

Recently, the medicinal aspects of various natural agents/substances have gained more public attention. Those include herbs, mushrooms, flowers, fruits, plant seeds, sea weeds, algae, tea, bark, shark cartilage and so on. We were particularly interested in one of well-established medicinal mushroom called *Phellinus linteus* (PL). Originally, PL has been used in Asian countries for centuries to prevent/treat ailments such as gastroenteric dysfunction, diarrhea, hemorrhage, rheumatoid arthritis, and cancers [7]. To develop it for anticancer therapeutics, different “PL-fractions”, i.e. bioactive extracts of PL, have been isolated and intensively studied. A number of studies have revealed that they had the antitumor, immunomodulatory, anti-angiogenic, and antioxidant properties [8-11]. In fact, antitumor/anticancer activities of those fractions have been demonstrated in various cancers/tumors *in vitro* and *in vivo* including prostate cancer, lung cancer, colon cancer, breast cancer, melanoma, leukemia, etc. [12-18]. Accordingly, we investigated if two distinct PL-fractions, PL-ES and PL-I-ES, might have potential anticancer effects on a variety of human cancers *in vitro*. In addition, a possible anticancer mechanism and induction of apoptosis by these fractions were also explored. More details are described and the significant findings are also discussed herein.

## Materials and Methods

### Cell culture

The 10 human cancer cell lines, PC-3 (prostate cancer metastasized to bone), DU-145 (prostate cancer metastasized to brain), and LNCaP (prostate cancer metastasized to lymph node), bladder cancer T24, kidney cancer ACHN, lung cancer A549, breast cancer MCF-7, stomach cancer AGS, liver cancer HepG2, and brain cancer U-87 cells, were obtained from the American Type Culture Collection (ATCC; Manassas, VA, USA) and employed in this study. All cancer cell lines were adapted to RPMI 1640 medium from their recommended media to rule out potential effects of various media on the experiment outcomes. RPMI 1640 medium also contained 10% fetal bovine serum, penicillin (100 U/mL), and streptomycin (100 µg/mL). Routinely, culture medium was changed every 3 - 4 days and the passage of cells was performed weekly. Two PL-fractions, PL-ES and PL-I-ES, were a gift from the manufacturer (Mushroom Wisdom, Inc., East Rutherford, NJ, USA). For experiments, cells were seeded in six-well culture plates at the initial cell density of 1 or 2 × 10^5^ cells/mL (depending on the cancer cell types) and were cultured with the varying concentrations of PL-ES or PL-I-ES. Cell morphology was monitored everyday and cell number/viability was assessed at 72 h by MTT (3-[4,5-dimethylthiazol-2-yl]-2,5-diphenyl-tetrazolium bromide) assay.

### MTT assay

Cell viability/growth was determined by the MTT assay following the vendor’s protocol (Sigma-Aldrich, St. Louis, MO, USA). Briefly, at the harvest time, 1 mL of MTT reagent (1 mg/mL) was added to each well in the six-well plate, followed by 3-h incubation at 37 °C. After removing MTT reagent, dimethyl sulfoxide (DMSO) was added to each well and absorbance of fomazan solution was read on a microplate reader. Cell growth was then expressed as the percent (%) of viable cells relative to the control reading (100%).

### Lipid peroxidation (LPO) assay

The severity of oxidative stress was assessed by the LPO assay measuring the formation of malondialdehyde (MDA), an end product from peroxidation of polyunsaturated fatty acids in the plasma membrane [19]. The amount of MDA formed will indicate the severity of oxidative stress: the more MDA formed, the greater oxidative stress. The detailed procedures were described in the vendor’s protocol (Abcam, Cambridge, MA, USA), and the amounts of MDA formed were measured by µM and converted to arbitrary values relative to controls (1.0).

### Enzymatic assays for caspase-3 (Csp-3) and caspase-9 (Csp-9)

Enzymatic activities of Csp-3 and Csp-9 were determined following the colorimetric method described in the manufacturer’s protocol (BioVison, Inc., Milpitas, CA, USA). Control or treated cells (about 1 × 10^6^ cells) were first lysed in lysis buffer by incubating on ice for 10 min. The supernatant (cell lysate) was collected by centrifugation at 13,000 rpm for 10 min and protein concentration was determined using Protein Assay Reagent (Pierce, Rockford, IL, USA) on a spectrophotometer. An equal amount (100 µg) of cell lysates was incubated with the reaction buffer containing dithiothreitol and the substrates, DEVD-pNA (for Csp-3) or LEHD-pNA (for Csp-9), at 37 °C for 2 h. The optical density of the reaction mixture was determined spectrophotometrically at 405 nm using a 96-well microplate reader. Fold-increase in Csp-3/9 activities was calculated by comparing the readings of treated samples with the level of the untreated control.

### Statistical analysis

All data were presented as mean ± standard deviation (SD), and statistical differences between groups were assessed with either the unpaired Student’s *t*-test or one-way ANOVA analysis. Values of P < 0.05 were considered to indicate statistical significance.

## Results

### Anticancer effects of PL-ES or PL-I-ES on various cancer cells

To assess anticancer effects of PL-ES or PL-I-ES on 10 different human cancer cells, a pilot study was first performed to find their effective concentrations (testing 0 - 700 µg/mL). Such a study indicated that PL-ES at 100 µg/mL and PL-I-ES at 100 - 250 µg/mL were significantly effective on these cancer cells (data not shown), so that the following studies were carried referring to this finding.

**Figure 1:**
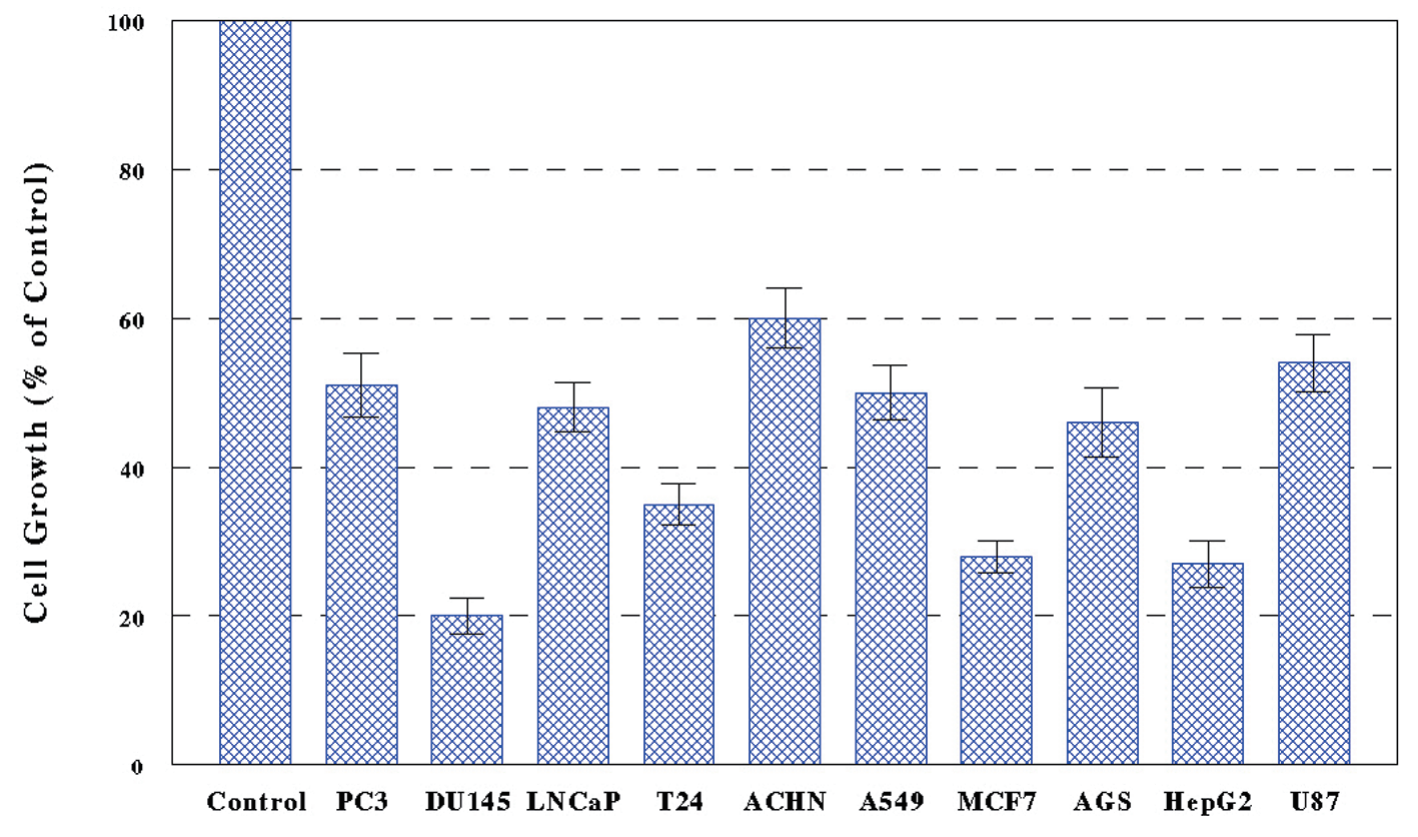
Effects of PL-ES on cell growth. Ten cancer cell lines, PC-3, DU-145, LNCaP, T24, ACHN, A549, MCF-7, AGS, HepG2, and U-87, were cultured with PL-ES (100 µg/mL) for 72 h and cell growth was assessed by MTT assay as described in Materials and Methods. Cell growth was expressed by the percent (%) of the reading of viable cells relative to controls (100%). Differences in cell growth between control and all treated cells are statistically significant (P < 0.05). All data are mean ± standard deviation (SD) from three separate experiments.

Ten cancer cells, PC-3, DU-145, LNCaP, T24, ACHN, A549, MCF-7, AGS, HepG2, and U-87, were treated with PL-ES (100 µg/mL) or PL-I-ES at 100 or 250 µg/mL, and cell growth/viability was determined in 72 h by MTT assay. The results showed that PL-ES (100 µg/mL) was highly potent inducing a 40-80% growth reduction in all 10 cancer cells (Fig. 1). It should be noted that PL-ES (250 µg/mL) tested in a pilot study had severe effects resulting in nearly complete (> 95%) cell death (data not shown).

**Figure 2:**
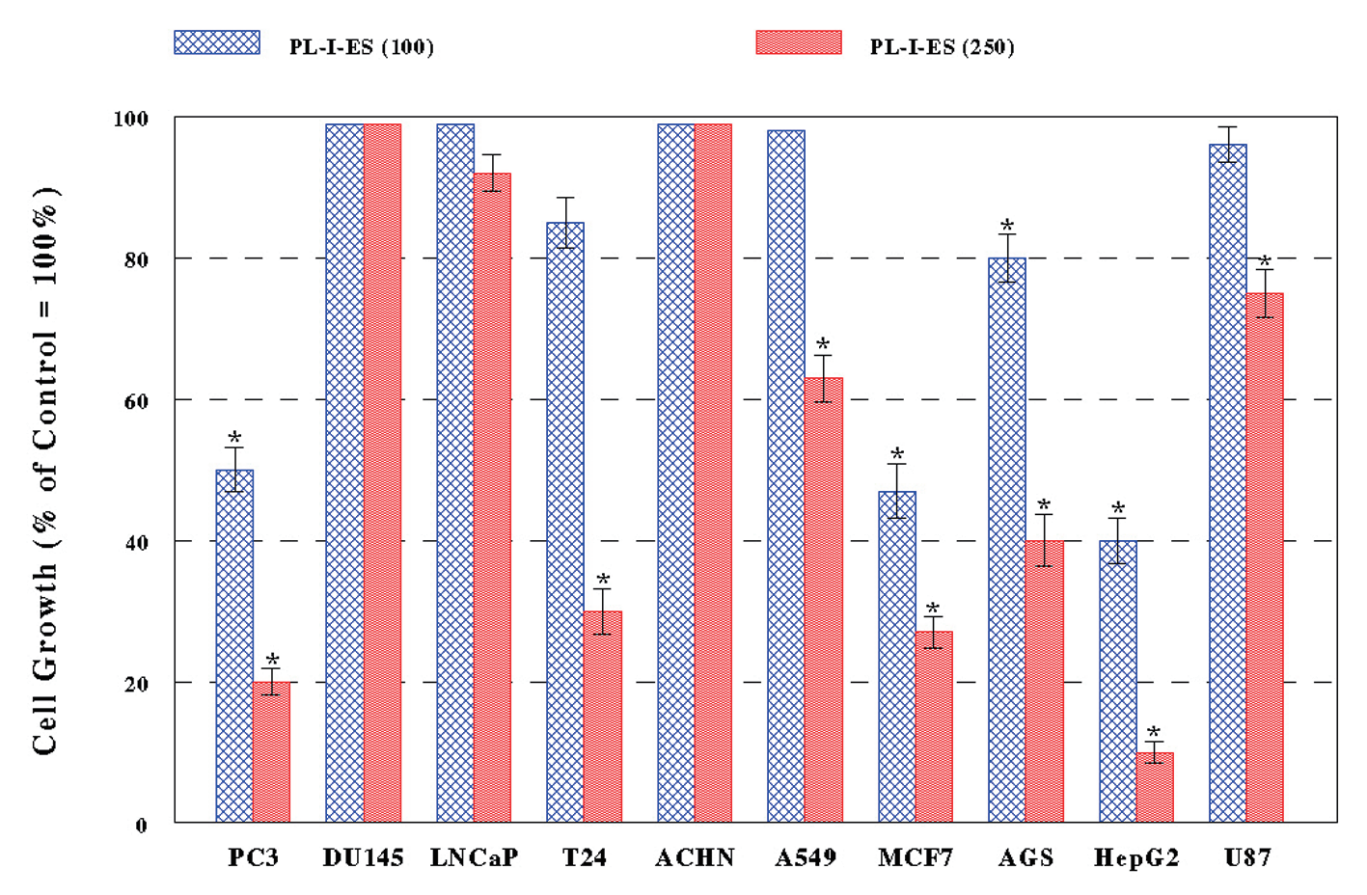
Effects of PL-I-ES on cell growth. Ten different cancer cells were cultured with PL-I-ES at either 100 or 250 µg/mL for 72 h and cell growth was determined by MTT assay. Cell growth was expressed by the percent (%) relative to controls (100%) (*P < 0.05). The data are mean ± SD from three independent experiments.

PL-I-ES (100 µg/mL) showed a 20-60% growth reduction in four cancer cells (PC-3, MCF-7, AGS, and HepG2) but had little effects on the rest of six cancer cells (Fig. 2A). In contrast, PL-I-ES (250 µg/mL) led to a 25-90% growth reduction in seven cancer cells, except for three cancer cells (DU-145, LNCaP, and ACHN) (Fig. 2B). This apparently shows that 250 µg/mL of PL-I-ES is significantly more effective than 100 µg/mL. Thus, these results suggest that both PL-ES and PL-I-ES have anticancer effects (reducing/inhibiting cell growth) but PL-ES appears to be more potent than PL-I-ES. Based on this finding, PL-ES at 100 µg/mL and PL-I-ES at 250 µg/mL were used in the rest of our study.

### Cytotoxicity through oxidative stress exerted by PL-ES or PL-I-ES

Those treated cells (with PL-ES or PL-I-ES) exhibited certain morphological changes, e.g., floating (due to a loss of cell anchorage) or cytoplasmic condensation, indicating that they were dying or dead due to cytotoxic effects. As it was possible that oxidative stress (with generation of free radicals) could exert such a cytotoxic effect, this possibility was then tested by LPO assay to assess the severity of oxidative stress exerted by PL-ES or PL-I-ES.

**Figure 3:**
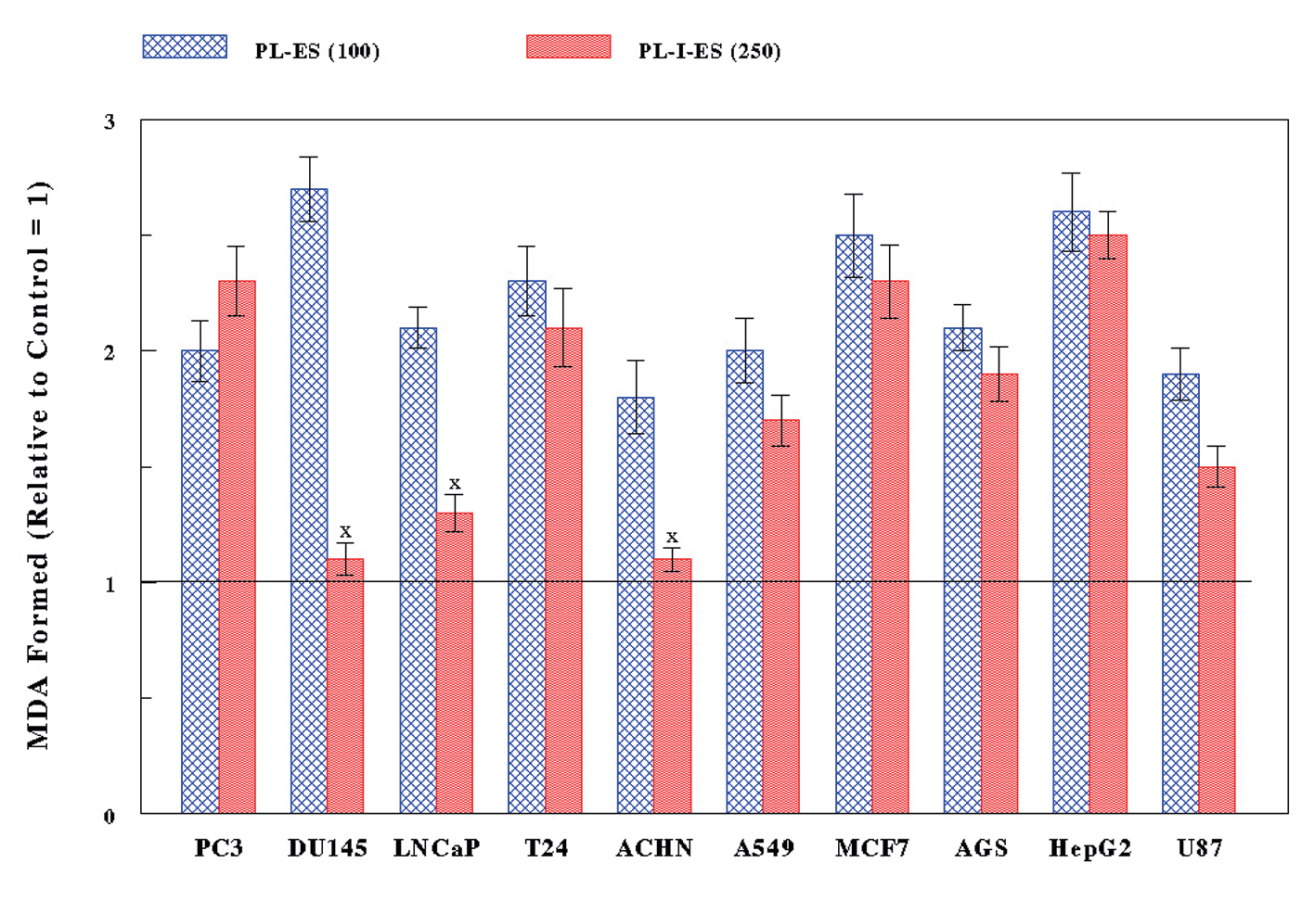
Exertion of oxidative stress. Ten cancer cells were treated with either PL-ES (100 µg/mL) or PL-I-ES (250 µg/mL) for 24 h and the severity of oxidative stress was assessed by LPO assay. The amounts of MDA formed indicate the degrees of oxidative stress, which are expressed arbitrarily relative to their respective controls (1.0 shown as a line). The amounts of MDF formed with PL-ES in all cancer cells and with PL-I-ES in seven cancer cells, except for three cancer cells (^X^P > 0.05), show statistically the significant differences (P < 0.05). All data represent mean ± SD from three separate experiments.

All 10 cancer cells were treated with PL-ES (100 µg/mL) or PL-I-ES (250 µg/mL) for 24 h and subjected to LPO assay to measure the amount of MDA formed: the more MDA formation, the greater oxidative stress. PL-ES (100 µg/mL) led to 1.8- to 2.7-fold more MDA having been formed in all cancer cell types compared to controls (Fig. 3). Meanwhile, PL-I-ES (250 µg/mL) showed the 1.5- to 2.5-fold increases in MDA formation in seven cancer cells but had little effects on three other cancer cells (Fig. 3). As a result, such significant increases in MDA formation (i.e. greater oxidative stress) by two fractions appear to be well correlated with the decreased/reduced cell growth. Therefore, these findings suggest that the growth reduction induced by PL-ES or PL-I-ES may at least result from cytotoxicity through oxidative stress.

### Possible induction of apoptosis by PL-ES or PL-I-ES

Strictly speaking, the growth reduction (induced by PL-ES or PL-I-ES) would be more appropriately described as the reduced “cell viability” (instead of growth). Along with morphological changes mentioned above, MTT assay primarily assesses cell viability - how many cells are viable/alive (or dead) - that will ultimately determine the status of cell growth such as the growth reduction. In addition, it is feasible that augmented oxidative stress may also ultimately induce cell death. Hence, the resulting growth reduction is more likely due to cell death or apoptosis (programmed cell death), which will eventually lead to the reduction in cell viability.

Accordingly, the possibility that the reduced cell growth or viability by two fractions could be attributed to apoptosis was examined next. Csp-3 and Csp-9 are the specific biochemical parameters known to play a central role in apoptosis and act as the positive or pro-apoptotic regulators: greater Csp-3/9 activities will greatly induce or promote apoptosis [20, 21]. All cells treated with PL-ES (100 µg/mL) or PL-I-ES (250 µg/mL) for 72 h were subjected to Csp-3/9 enzymatic assay as described in Materials and Methods. The results are then summarized in Table 1. In the case of PL-ES, both Csp-3 and Csp-9 in all 10 cancer cells were significantly (P < 0.03) activated by 2.6 - 3.5 and 3.0 - 4.4 folds, respectively, compared to those in control (untreated) cells. On the other hand, with PL-I-ES, Csp-3 was significantly (P < 0.05) activated by 2.3 - 3.7 folds (compared to controls) in seven cancer cells (PC-3, T24, A549, MCF-7, AGS, HepG2, and U-87), and Csp-9 was also activated by 2.6 - 4.5 folds in the same seven cancer cells. Overall, PL-ES appears to be capable of more potently activating Csp-3/9 than PL-I-ES, and such activation of two enzymes is well associated with the reduced cell growth/viability, due to induction of apoptosis. Therefore, this study suggests that PL-ES and PL-I-ES may induce apoptosis (through oxidative stress), most likely accounting for the significant growth/viability reduction.

**Table 1:** Effects of PL-ES or PL-I-ES on Caspase Activity

Cancer cells (controls)	PL-ES (100 µg/mL)		PL-I-ES (250 µg/mL)	
	Csp-3	Csp-9	Csp-3	Csp-9
PC-3 (1.0)^a^	3.0	3.4	3.5	4.2
DU-145 (1.0)^a^	3.5	4.4	1.2^b^	1.3^b^
LNCaP (1.0)^a^	2.9	3.3	1.5^b^	1.6^b^
T24 (1.0)^a^	3.3	4.2	3.2	3.9
ACHN (1.0)^a^	2.6	3.0	1.1^b^	1.3^b^
A549 (1.0)^a^	2.8	3.3	2.5	2.9
MCF-7 (1.0)^a^	3.4	4.2	3.3	4.0
AGS (1.0)^a^	2.9	3.2	2.9	3.5
HepG2 (1.0)^a^	3.4	4.3	3.7	4.5
U-87 (1.0)^a^	2.8	3.1	2.3	2.6

^a^A value of 1.0 in parenthesis indicates an arbitrary number set for activity of caspase-3 (Csp-3) and caspase-9 (Csp-9) in control or untreated cells, following the results of enzymatic assays. ^b^P > 0.05 (differences are not significant compared to controls).

## Discussion

Because of ineffective therapeutic options and unsatisfactory outcomes, a variety of cancer patients and their families are desperately seeking and longing for a better, more effective therapeutic modality. We have been exploring an alternative or unconventional way to improve the therapeutic efficacy using natural substances or products. Among them, we have come across the bioactive mushroom extracts, PL-ES and PL-I-ES, isolated from PL and whether these two extracts would have anticancer effects on various cancer cells was investigated herein.

PL-ES (100 µg/mL) exhibited potent anticancer activity, leading to a significant (40-80%) growth reduction in all 10 cancer cells. Although PL-I-ES (100 µg/mL) was effective on only four cancer cells, PL-I-ES (250 µg/mL) has significantly (25-90%) reduced the growths of seven cancer cells. Thus, PL-ES appears to be more potent than PL-I-ES that requires the relatively higher concentration (250 µg/mL) to be effective.

To have a better understanding of how two fractions can induce the significant growth reduction, whether oxidative stress could be involved or play a key role was examined. LPO assays revealed that two fractions indeed increased MDA formation, indicating exertion of oxidative stress. PL-ES (100 µg/mL) led to the significant increase in MDA formation in 10 cancer cells, while seven cancer cells showed such an increase with PL-I-ES (250 µg/mL). Nevertheless, the key point is that such an increase in MDA formation or greater oxidative stress appears to have a good correlation with the reduced cell growth. In other words, the growth reduction (due to cell death) induced by PL-ES or PL-I-ES could be attributed to the cytotoxic effect of oxidative stress.

However, there is some concern that oxidative stress (exerted by two fractions) is cytotoxic and harmful to cancer cells but could be also harmful to normal (non-cancerous) cells. This may raise a question on their possible clinical or therapeutic utility because of the non-specific, cellular destructive nature of free radicals. However, cancer cells have been shown to be more vulnerable to free radicals than normal cells [22]. It is not fully understood but several evidences yet suggest that the inherent difference in activity or presence of antioxidant enzymes between cancer and normal cells may account for such different susceptibility to free radicals. For instance, two potent antioxidant enzymes, catalase and glutathione peroxidase, have been found to be often deficient or have significantly lower activities in cancer tissues than in normal counterparts [23]. It is thus possible that the amount of free radicals generated by two fractions could be cytotoxic enough to kill cancer cells (due to a low antioxidant enzyme activity) but would not be enough to even harm normal cells (with a high enzyme activity). Further studies for analyzing certain antioxidant enzymes in 10 cancer cell lines are currently underway.

It was also interesting and critical to assess if the growth/viability reduction by PL-ES or PL-I-ES might be linked to apoptosis. This possibility was tested by measuring enzymatic activities of Csp-3 and Csp-9, the positive apoptotic regulators [20, 21]. As both Csp-3 and Csp-9 were significantly activated by PL-ES or PL-I-ES, the reduced growth reduction appears to be more likely attributed to apoptotic cell death. Therefore, it is plausible that these two fractions are capable of commonly inducing apoptosis in a variety of cancer cells, accounting for their anticancer activities.

Lastly, regarding the safety of PL-ES or PL-I-ES, they are quite safe to be used even in a therapeutic purpose since they originally come from a non-poisonous mushroom (PL). In addition, they have been consumed dietetically or medicinally by Japanese people for hundreds of years and still contribute to an important part of Japanese daily life. Nevertheless, prior to a possible clinical trial, the safety, efficacy, and potential adverse effects of these fractions must be closely and cautiously monitored or assessed in animal study. Such study is currently in progress.

In conclusion, two bioactive extracts of PL, PL-ES and PL-I-ES, demonstrated their anticancer activities on 10 different human cancer cell lines *in vitro*. Such anticancer activity was presumably mediated through oxidative stress, which could ultimately induce apoptosis. Therefore, they appear to be promising and may provide a safer and more effective therapeutic modality for a variety of human malignancies. Further studies are warranted.
